# Construct validity and internal consistency of the Home and Family Work Roles Questionnaires: a cross-sectional study with exploratory factor analysis

**DOI:** 10.1186/s12905-023-02199-1

**Published:** 2023-02-10

**Authors:** A. Dabbagh, H. Seens, J. Fraser, J. C. MacDermid

**Affiliations:** 1 Health and Rehabilitation Sciences, Faculty of Health Sciences, Western University, London, ON Canada; 2 Collaborative Program in Musculoskeletal Health Research, Bone and Joint Institute, Western University, London, ON Canada; 3 Windsor University School of Medicine, Cayon, St. Kitts and Nevis; 4 Department of Physical Therapy, Faculty of Health Sciences, Western University, London, ON Canada; 5 Department of Computer Science, University of Guelph, Guelph, ON Canada; 6 Physical Therapy and Orthopedic Surgery, Western University, London, ON Canada; 7 Roth|McFarlane Hand and Upper Limb Centre, St. Joseph’s Health Centre, London, ON Canada

**Keywords:** Family work, Unpaid work roles, Wwomen work, Home responsiblities, Exploratory factor analysis

## Abstract

**Introduction:**

Exploratory Factor Analysis (EFA) measures the underlying relationships between questionnaire items and the factors (“constructs”) measured by a questionnaire. The Home and Family Work Roles Questionnaire has not been assessed using EFA; therefore, our objective was to identify the factors measured by this questionnaire.

**Methods:**

We recruited 314 persons to complete the questionnaire and to answer several demographic questions. We determined if the data was factorable by performing Bartlett’s test of sphericity and the Kaiser–Meyer–Olkin measure of sampling adequacy. We used the Factor package in Jamovi statistical software to perform EFA. We employed an Oblimin rotation and a Principal Axis extraction method. We also calculated the internal consistency of the questionnaire as a whole as well as each individual question.

**Results:**

Our sample consisted of 265 (85%) women, 45 (14%) men, and 3 (1%) non-binary or other genders. The mean age of our participants was 34.65 (SD = 11.57, range = 18–65) years. EFA suggested a three-factor model. Questions 11, 13, 14, 15, and 16 measured one factor (we interpreted this as “Caregiving Roles”), questions 1, 3, 4, 8, 9, 10, 18, and 19 measured a different factor (“Traditionally Feminine Roles”), and questions 2, 5, 6, and 12 measured the “Traditionally Masculine Roles”. The questionnaire and each individual question demonstrated excellent internal consistency (Cronbach's α > 0.90).

**Conclusion:**

The Home and Family Work Roles Questionnaire may measure three distinct factors, which we have named Caregiving, Traditionally Feminine, and Traditionally Masculine Roles. This aligns with the theory used in developing the questionnaire. Separation of the Home and Family Work Roles Questionnaire into three sub-scales with distinct scores is recommended to measure each of the recommended constructs.

**Supplementary Information:**

The online version contains supplementary material available at 10.1186/s12905-023-02199-1.

## Introduction

The changing role of women across the globe has been observed in many countries in the last century, as women are taking up more prominent roles and responsibilities in the government, politics, and all levels of the society [1–4]. Historically and culturally, women had concerned themselves with more household tasks compared with men [1, 5]. Measuring whether this changing role of women in the society is accompanied by the changes in the home and family work responsibilities is important in determining many factors such as the roots of many family conflicts, couples’ agreement or discordance, domestic violence, family’s psychosicial needs, among others [6]. To measure the proportion of home and family work roles done by each partner, it is imperative to have systematic validated measurement tools.

The Home and Family Work Roles Questionnaire was developed by Dr. Joy MacDermid in 2018 [7]. This questionnaire asks about the type and proportion of work an individual performs related to tasks needed to care for the home or family [7]. The questionnaire developer proposed that some items are ‘gendered’ role assignments in daily household tasks, and some items indicate a ‘caregiving’ role, which could potentially be used to explore how typically gendered tasks are distributed amongst family members [7]. The proposed ‘typically masculine gendered’ items were outdoor cleaning, home repairing, lawn mowing and snow shovelling, and vehicle maintenance [7]. The ‘typically feminine gendered’ items related to indoor cleaning, laundry, house decorating, and arranging family appointments and activities [7]. Lastly, the ‘caregiving’ items were caring for sick children and ill or elder adult families, taking families to their activities or appointments, and helping children with homework [7].

Two scoring methods were originally proposed for this questionnaire, which were item scoring (where scores range from zero to ten for each question) and percentage scoring (where the scores range from zero to 100% for each question) [7]. The item scoring system indicates the amount of work being done, whereas the percentage scores are more useful when looking at the proportion of the family work role responsibilities performed by an individual.

The data from the Home and Family Work Roles Questionnaire can serve multiple purposes [8, 9]. Firstly, the item percentages listed in the questionnaire indicate the proportion of the workload of the house in that area. Further, the mean of the items can be used to calculate the overall proportion of the workload being done by an individual. The total sum of family role responsibilities work is calculated by adding up all the items (with the maximum score of 10 for each item) and then divided by 180 (the total possible score). Further, it is possible to calculate the amount or proportion of the masculine, feminine, or caregiving subset workloads separately by adding up the items relating to them [9]. This data can be used for individuals or to calculate the couple's agreement or discordance scores when a couple completes the questionnaire.

Exploratory Factor Analysis (EFA) is founded on philosophical and statistical principles [10]and was first applied in 1904 by Spearman [11]. Soon after, it was rapidly adopted by many researchers to evaluate theories and validate measurement instruments [12]. This statistical method is used to identify the latent variables (also known as constructs, factors, dimensions, or internal attributes) that can parsimoniously demonstrate the covariation of a set of measured variables [13]. In simpler words, EFA measures the factors that clarify the structure and order of the measurement instruments [13].

The Home and Family Work Roles Questionnaire had not been subjected to EFA; hence its construct validity has not been previously established. It is important to establish the underlying factors within this questionnaire to measure different constructs as intended by this questionnaire. Therefore, the primary objective of this study was to undertake EFA to identify the factors measured by the Home and Family Work Roles Questionnaire. The secondary objective was to assess the internal consistency of the items in this questionnaire.

## Materials and methods

### Study design and ethics

This was a cross-sectional observational study to quantitatively assess the factors measured by the Home and Family Work Roles Questionnaire. Western University’s Health Sciences Research Ethics Board (HSREB) approved this study on June 25, 2020, with project ID number 115790. All study participants provided informed consent prior to participation.

### Participants and recruitment

Study participants were selected from a larger sample of data collected through a survey designed to assess changes in mental health in terms of family responsibilities, depression, and substance use following the start of the COVID-19 pandemic [14]. For this study, we only used the data related to the Home and Family Work Roles Questionnaire, as well as some descriptive measures, as explained in the following section. Anyone aged 18 years old or older who was able to read and understand English was eligible to participate. The participant recruitment took place from June to August 2020 and the questionnaire was completed using Qualtrics. We reached participants by sharing study ads on multiple social media platforms such as Facebook and Instagram.

### Measures

#### Home and Family Work Roles Questionnaire

Given our purpose to validate the Home and Family Work Roles Questionnaire, this questionnaire was the main measure of our study. The instruction was: “Think about the work you did to take care of your home and family AFTER the COVID-19 pandemic (after March 11, 2020). Please do not count the work done by anyone else (family, friends, spouses, paid staff, etc.). Slide the scale to represent the percentage of the work you did. If the question does not apply to you (for example: you do not have children), then choose not applicable.” The questionnaire consisted of 19 questions, which asked about house cleaning, indoor cleaning, laundry, home decorating, home repairs, lawn mowing, gardening, preparing meals, grocery and supplies shopping, driving family to appointments and activities, arranging family appointments and activities, maintaining vehicles, helping or supervising children with homework, caring for children in the home or when sick, caring for other family members, earning family income, managing family finances and bills. The response options were in percentages, starting from 0% (with 10% increments) and ending with 100%.

#### Descriptive measures

To assess participants’ demographics, we assessed multiple descriptive measures. (A) Current employment status, with the response options being paid employees, self-employed, laid off, stay-at-home parent/caretaker, student, retired, unable to work due to a disability, and other. (B) Paid job status prior to the pandemic, with four response options: yes, full-time (more than 20 h/week), yes, part-time (20 or fewer hours/week), no, and other. (C) Paid job status since the pandemic started, with the same response options as the previous question. (D) Working from home as a result of the pandemic, with the response options being with a similar workload, with a greater workload, with a lighter workload, I still go into my workplace, I lost my job, and I have always worked from home. (E) Marital status, with response options of single, common-law, married, divorced, widowed, and other. (F) Gender of the participant and their partner, with response options of man, woman, non-binary, agender, and other. (G) Age, measured on a continuous scale. (H) Sex of the participant, with response options of male, female, and other. (I) The number of people in the household. (J) Ethnic origin, with response options being North American aboriginal, Black, White, Arab, Latin, Central, and South American, South Asian (e.g., East Indian, Pakistani), West Asian (e.g., Iranian, Afghan), Southeast Asian (e.g., Vietnamese, Cambodian), East Asian (e.g., Chinese, Japanese, Korean), Pacific Islands (e.g., Fijian, Hawaiian), and other.

### Statistical analyses

#### Descriptive statistics

We performed all the statistical analyses using Jamovi Statistical Software Version 1.6.23.0 [15]. We used the ‘Exploration’ function to measure the descriptive statistics for all the descriptive measures.

#### Checking the assumptions of EFA

Before submitting the data for EFA, we checked the EFA assumptions to ensure that the correlation matrix was factorable. Firstly, Bartlett’s test of sphericity [16] was used and indicated that the correlation matrix was not random. Then, we applied the Kaiser–Meyer–Olkin measure of sampling adequacy, which was required to be above 0.5 for all variables [17].

#### Examining the construct validity: exploratory factor analysis

After establishing the assumptions of EFA, three- and two-factor solutions were sequentially examined for their adequacy. The EFA was conducted with Jamovi statistical software and its *Factor* package [15]. We employed an Oblimin rotation because we assumed that due to the nature of the Home and Family Work Roles Questionnaire, the factors would be correlated; hence an oblique rotation method (Oblimin) was deemed appropriate [18]. We extracted data using the *Principal Axis* extraction method as it is the most appropriate method to recover the relatively weak factors when the assumptions of normality are violated [19].

#### The number of factors retained in the model

Several studies recommend using a combination of different criteria, as well as the theory, to decide on the number of factors to retain in the model [13, 20]. In this study, we used three approaches to determine the number of factors to retain in the model, as explained below.

**Eigenvalue method**. This is the most common approach to determining the number of factors to retain in the model. We followed the recommendation by Kaiser [21] to retain any factors with an eigenvalue of above one.

**Visual scree plot**. This is a graphical method consisting of the eigenvalues and the factors [20]. We retained any factors above the distinct point of break in the slope of the scree plot (also known as the point of inflection or elbow point), as recommended by several studies [13, 22].

**Parallel analysis**. This statistical method generates a random set of data, simulating the same number of variables and participants as the real data [13].

In addition to these statistical methods, we also considered the underlying theory in developing the Home and Family Work Roles Questionnaire. Based on the theory, three original subsets of questions (or factors) exist in this questionnaire, which are related to masculine, feminine, and caregiving roles.

#### Factors’ adequacy

We established a priori criteria for determining factors’ adequacy. According to our sample size, a factor loading of at least 0.37 was considered both practically and statistically salient [23]. Further, to honor the simplicity of the final structure, we rejected complex loadings that were salient on more than one factor [24]. After applying these criteria, in order to maintain transparency we presented the factors loadings in the results table. Given this, factors were considered adequate when they (1) had at least three salient loadings that were equal to or above 0.37, (2) were theoretically meaningful, and (3) had internal consistency of at least 0.70.

#### Examining gender differences between factors

We ran independent samples t-test to compare the means of each question for women and men. For these analyses, we had to remove three participants who had identified their gender as non-binary or other because the number of respondents did not reach the minimum power required to perform an analysis. For each of the t-tests, one of the questions was the dependent variable, and gender (at two levels: woman and man) was the grouping variable. We reported t-test statistics, *p*-value, degrees of freedom, mean difference, standard error difference, 90% confidence intervals, and effect size.

#### Examining reliability: internal consistency

We examined both the scale reliability statistics and the item reliability statistics and reported Cronbach's α. We followed the recommendations of Nunnally, where Cronbach's α of above 0.70 is considered acceptable for research purposes, and scores above 0.90 are considered excellent internal consistency [25].

#### Sample size

We included participants who responded to at least 16 questions (out of 19) of the Home and Family Work Roles Questionnaire on the online survey. Any participant with less than 16 responses was case-wise deleted. Our sample size was adequate for EFA for two reasons. Firstly, based on the rule of thumb method for sample size calculation, a ratio of 10 participants per question (10:1) is recommended [26], which is 190 (10*19) participants in our study. Secondly, based on the instructions provided by Norman and Streiner, “factor analysis is a large-sample procedure” and an arbitrary sample of 100 to 200 is recommended ([23], p. 223).

## Results

### Descriptive statistics

After removing participants who had more than three unanswered questions on the Home and Family Work Roles Questionnaire, we included 314 participants for the EFA analysis. Our sample consisted of 265 (85%) women, 45 (14%) men, and three (1%) non-binary or other genders. Further, there were 266 (86%) females, 43 (14%) males, and two (1%) participants identified as other sex. The gender of the partner was missing for 126 participants, and of those who responded, 156 (83%) had men partners, 31 (16%) had women partners, and one (1%) participant had a partner with non-binary gender.

The mean age of our participants was 34.6 (SD = 11.5, range = 18–65) years. Regarding marital status, 171 (54%) were married, 98 (31%) were single, 20 (6%) were divorced, 18 (6%) were in a common-law relationship, and six (3%) were in other types of relationships. For the number of people in the household, 131 (42%) participants reported four people, 72 (23%) reported three people, 62 (20%) participants reported five people, 33 participants reported more than five people, and 15 (4%) reported less than three people live in their household.

Regarding the ethnicity of the participants, the majority identified themselves as White (n = 226, 72%). Of the remaining participants, 15 (5%) were South Asian (e.g., East Indian, Pakistani), 15 (5%) were East Asian (e.g., Chinese, Japanese, Korean), 12 (4%) were West Asian (e.g., Iranian, Afghan), seven (2%) were Black, and the remaining 39 (12%) had other ethnicities such as Latino and Aboriginal.

In terms of their current employment status, 152 (48%) were paid employees, 71 (23%) were students, 29 (9%) were stay-at-home parents/caretakers, 21 (7%) had other current employment statuses, 20 (6%) were self-employed, 14 (4%) were laid off, five (2%) were unable to work due to disability, and two (1%) were retired.

As for their paid-job status pre-COVID-19 pandemic, 156 (50%) were working full-time, 75 (24%) did not have a paid job, 67 (21%) were working part-time, and 15 (5%) responded to other paid-job statuses pre-pandemic. Post-COVID-19 pandemic, the number of people who worked full-time had decreased to 129 (43%), the number of unemployed participants surged to 90 (30%), 56 (19%) were working part-time, and 27 (9%) responded to other. Lastly, for the question on working from home, 67 (36%) said they still go into their workplace, 56 (30%) were working from home with a similar workload, 34 (18%) with a greater workload, 11 (6%) with a lighter workload, 14 (8%) were always working from home, and three participants (2%) had lost their job.

### EFA results

The results of Bartlett’s test of sphericity indicated that the correlation matrix was not random, χ^2^(171) = 2120.19, *p* < 0.001. Further, the Kaiser–Meyer–Olkin measure of sampling adequacy was 0.90, which is well above the minimum sampling adequacy requirements for conducting EFA.

**Fig. 1:**
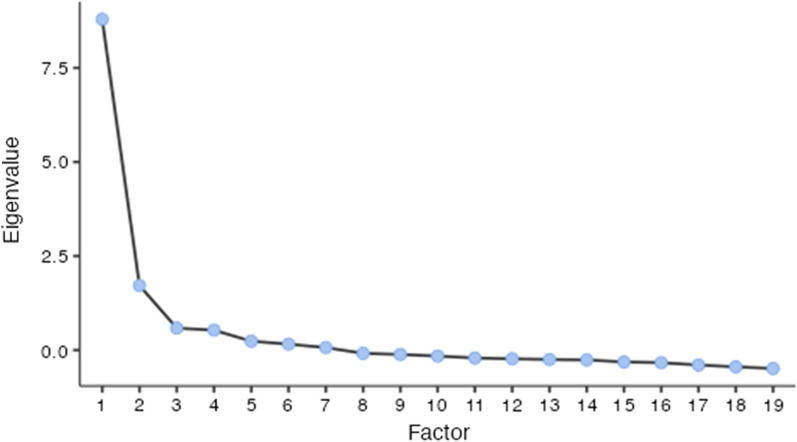
Scree plot

Parallel analysis, eigenvalue method, and scree plot (Fig. 1) suggested that two factors must be retained, but theory indicated that three factors were required. We decided to proceed with a three-factor solution as it yielded the most parsimonious model (Table 1).

**Table 1:** Exploratory factor analysis of the Home and Family Work Roles Questionnaire

Questions	Factor**s**			
	Caregiving roles	Traditionally feminine roles	Traditionally masculine roles	Uniqueness
Q1—House cleaning	0.15	**0.50**	0.18	0.48
Q2—Outdoor cleaning	0.13	0.02	**0.80**	0.25
Q3—Laundry	0.06	**0.66**	0.04	0.47
Q4—Home decorating	0.11	**0.56**	0.17	0.44
Q5—Home repairs	− 0.01	0.03	**0.81**	0.33
Q6—Mow lawn	− 0.13	− 0.05	**0.81**	0.44
Q7—Garden	0.21	0.12	**0.51**	0.51
Q8—Prepare meals	0.04	**0.73**	− 0.08	0.49
Q9—Shop for groceries and supplies	− 0.16	**0.80**	0.07	0.44
Q10—Drive family to appointments and activities	0.31	**0.45**	− 0.02	0.53
Q11—Arrange family appointments and activities	**0.54**	0.43	− 0.10	0.29
Q12—Maintain vehicles	− 0.01	0.11	**0.62**	0.53
Q13—Help children with homework	**0.94**	− 0.04	0.04	0.15
Q14—Supervise children with homework	**0.93**	− 0.01	− 0.01	0.16
Q15—Care for children in the home	**0.93**	− 0.03	0.06	0.13
Q16—Care for children when sick	**0.89**	0.08	− 0.01	0.11
Q17—Care for other family members	**0.32**	**0.35**	0.09	0.56
Q18—Earn family income	0.12	**0.43**	0.24	0.54
Q19—Manage family finances/bills	0.21	**0.44**	0.20	0.47

'Principal Axis' extraction method was used in combination with an 'Oblimin' rotation. Salient pattern coefficients ≥ .37 in boldface

All three factors had at least three salient loadings at values of 0.40 or greater. Considering the conceptual commonalities between the reasons (variables), we labelled factor one as Caregiving Roles, factor two as Traditionally Feminine Roles, and factor three as Traditionally Masculine Roles. The correlation of the factors was checked and established well below the threshold for high correlation. The highest observed correlation was between Caregiving and Feminine roles, with* r* = 0.71, which is consistent with the expectation that caregiving is a more traditionally feminine role. The remaining two correlations were below 0.70; therefore, one can conclude that the three factors were distinct from each other.

**1- Caregiving roles.** Questions 11, 13, 14, 15, and 16 were labelled as the caregiving roles. These questions related to arranging family appointments and activities, helping or supervising children with homework, care for children in the home or when sick. Additional file 1: Appendix I summarizes the descriptive statistics of the Caregiving Roles factors.

**2- Traditionally feminine roles.** Questions 1, 3, 4, 8, 9, 10, 18, and 19 were all referring to feminine work roles and responsibilities. These questions asked about house indoor cleaning, laundry, home decorating, preparing meals, shopping for groceries and supplies, driving family to appointments and activities, earning family income, and managing family finances or bills.

Question 17 (care for other family members, e.g., elderly) had almost equal loading (also known as cross-loading) on both the Caregiving and Traditionally Feminine Roles factors, 0.32 and 0.35, respectively. Hence, it was not categorized as either of these factors. Please refer to Additional file 1: Appendix II for the descriptive statistics of Traditionally Feminine Roles factor.

**3- Traditionally masculine roles.** Lastly, the third factor was labelled as Traditionally Masculine Roles and entailed questions 2, 5, 6, 7, and 12. These questions relate to outdoor cleaning, home repairs, lawn mowing, gardening, and maintaining vehicles. Descriptive statistics can be found in Additional file 1: Appendix III.

### Gender differences between factors

The results indicated that, except for questions 18 and 19 (earning family income and managing family finances/bills), there was a statically significant difference between men and women for all the remaining questions. For questions related to outdoor cleaning, home repairs, lawn mowing, and maintaining vehicles (Traditionally Masculine Roles factor), men had done a greater percentage of the family work (statistically significant) compared to women. For all the remaining questions (including Traditionally Feminine and Caregiving Roles factors), women had done more work (statistically significant) than men. The detail of these analyses can be found in Table 2.

**Table 2:** Independent samples t-test with gender as the grouping variable

Questions	Statistic	df	p	Mean difference	SE difference	Lower 95% CI	Upper 95% CI	Cohen’s d effect size
Q1—House cleaning	− 3.40	309.00	< 0.001	− 1.46	0.43	− 2.31	− 0.62	− 0.54
Q2—Outdoor cleaning	3.14	304.00	0.002	1.59	0.51	0.59	2.59	0.50
Q3—Laundry	− 6.12	309.00	< 0.001	− 2.93	0.48	− 3.88	− 1.99	− 0.98
Q4—Home decorating	− 2.81	276.00	0.005	− 1.81	0.64	− 3.08	− 0.54	− 0.48
Q5—Home repairs	4.31^a^	302.00	< 0.001	2.31	0.54	1.26	3.37	0.70
Q6—Mow lawn	6.96^a^	254.00	< 0.001	3.96	0.57	2.84	5.08	1.17
Q7—Garden	− 1.97	292.00	0.050	− 1.17	0.60	− 2.35	− 4.41	− 0.32
Q8—Prepare meals	− 5.01	309.00	< 0.001	− 2.30	0.46	− 3.20	− 1.40	− 0.80
Q9—Shop for groceries and supplies	− 2.07	309.00	0.039	− 1.18	0.57	− 2.30	− 0.06	− 0.33
Q10—Drive family to appointments and activities	− 2.75^a^	292.00	0.006	− 1.71	0.62	− 2.93	− 0.49	− 0.45
Q11—Arrange family appointments and activities	− 5.49^a^	296.00	< 0.001	− 3.52	0.64	− 4.78	− 2.26	− 0.90
Q12—Maintain vehicles	5.64^a^	294.00	< 0.001	3.14	0.56	2.05	4.24	0.93
Q13—Help children with homework	− 4.38^a^	279.00	< 0.001	− 2.82	0.64	− 4.09	− 1.55	− 0.73
Q14—Supervise children with homework	− 4.35^a^	273.00	< 0.001	− 2.89	0.67	− 4.21	− 1.58	− 0.73
Q15—Care for children in the home	− 4.77	302.00	< 0.001	− 2.72	0.57	− 3.84	− 1.59	− 0.77
Q16—Care for children when sick	− 5.38^a^	287.00	< 0.001	− 3.46	0.64	− 4.73	− 2.20	− 0.90
Q17—Care for other family members	− 2.60	243.00	0.010	− 1.57	0.60	− 2.76	− 0.38	− 0.45
Q18—Earn family income	1.07	288.00	0.286	0.65	0.61	− 0.54	1.84	0.18
Q19—Manage family finances/bills	− 1.41	301.00	0.160	− 0.92	0.66	− 2.22	0.37	− 0.23

^a^Levene's test is significant (p < 0.05), suggesting a violation of the assumption of equal variances. The mean differences with negative signs indicate that women had done more work compared with men

These were based on independent samples t-test for each question to compare the mean score differences between men and women. The underlying assumptions for normality and homogeneity of variance were violated for some of the questions. However, we decided to employ this test because most items were supported (could put the number supported here too) and since this test has a high tolerance for non-normality in samples higher than 25 in each group.

### Internal consistency

The overall Cronbach’s α of the questionnaire was 0.94, indicating excellent internal consistency. When items were dropped one at a time, the overall Cronbach’s α remained at 0.93 to 0.94; hence, no item reduction was required.

## Discussion

We conducted an EFA to determine the construct validity of the Home and Family Work Roles Questionnaire and to assess the number of latent factors it entails. Our research identified that this questionnaire appears to measure three distinct factors: Caregiving Roles, Traditionally Feminine Roles, and Traditionally Masculine Roles. Our results align with the theoretical objectives in the designing and development of this questionnaire, which suggested the existence of three subsets of factors. These results are the first to explore the validity of using the Home and Family Work Roles Questionnaire as a self-reported assessment of family work roles and responsibilities among a large sample of 314 participants. Family work role, in contrast to employment status, is unpaid work that is often not measured or considered in health research.

The results of the EFA indicated strong support for a three-factor solution comprising Caregiving Roles, Traditionally Feminine Roles, and Traditionally Masculine Roles. Further, by using a conservative correlation threshold of 0.37 to determine factor loading, we categorized 18 questions into three latent factors without confounding or overlap. The correct number of factors to retain in the model, resulting in a model that is parsimonious yet comprehensible, is one of the most important decisions in EFA [13]. Extracting too many or too few factors would lead to potential undesirable error variance or leaving out valuable common variance [20]. In our EFA, even though we had two factors with Eigenvalues above one, we decided to also retain the third factor with an Eigenvalue below one due to its conceptual validity. All the latent factors were loaded by at least four salient loadings, and this three-factor solution led to the most parsimonious model and was supported by theory in the development of the questionnaire.

When looking at the amount of work done by women and men, women outperformed men in tasks relating to housekeeping work, and caregiving to children and other family members. With respect to earning family income and managing family finances, women and men had a similar contribution. In a 2016 study [27] that looked at attitudes towards women’s work and family roles in the United States from 1976 to 2013, a major shift towards more egalitarian gender roles was identified. Yet, our results indicate that women not only made a higher contribution to traditionally feminine tasks and caregiving tasks compared with men, but also made relatively large contributions in earning family income. Higher contribution of women in earning family income might reflect women’s changing role in society where they have access to the labour force. However, this does not seem to be associated with more equal division of family role tasks.

In the subcategory of the questions labelled as Caregiving Role, women had done significantly more work compared with men in all tasks, and the effect sizes were large. Only one question (question 17: care for other family members) had similar cross-loading on feminine and caregiving role factors; meaning that unlike other caregiving roles that had strong loading on Caregiving Roles factor, caring for other family members could also be considered part of feminine roles and responsibilities. Upon examining the amount of work done within this task between genders, even though women had done a greater proportion (statistically significant) of the family work compared with men, the mean difference was not as large as other caregiving tasks and the effect size was small to medium [28]. This suggests that within the caregiving roles, the only task that men had shown some contribution was caring for other family members. This might be attributable to care in this case being interpreted as emotional caring, whereas the other items more clearly positioned care as tangible activities in caring for specific family members. Another potential explanation is that some caring activities may be related to the person for whom the care is being provided. Men may provide more care for elder or sick people from the man’s family side live in the household.

Our study categorized earning family income as a Traditional Feminine Role factor. Although men had done more work in this task compared with women, the mean difference was small, and the difference was not statistically significant. Earning family income was traditionally considered a gendered expectation for men, as more families were “head-complement” family type where women do not work outside the home [29]. Women joined the labor force in large numbers during the first world-war and then increasingly after 1980, creating a shift towards career-earner families (where men have a career and women have a job), and two-career families (where both partners are employed in career positions) was observed [29, 30]. As a result, the attitude towards men being the sole “economic-provider” and working outside the home being a gendered role for men changed [31]. The impact of this shift in men and women’s gendered tasks which was compounded by the COVID-19 pandemic, where more men might have lost their jobs when participating in this survey, the factor loading was stronger on Traditionally Feminine Roles factor (0.43) than the Traditionally Masculine (0.24).

Internal consistency reliability assessment suggested that all 19 questions of the Home and Family Work Roles Questionnaire measured the same construct and that there was no indication that any of the questions should be excluded. The Cronbach’s α of 0.94 indicates excellent reliability [25] when using this questionnaire as a screening tool or as an outcome measure.

It is difficult to draw comparisons between the Home and Family Work Roles Questionnaire and other questionnaires as no other questionnaire exists that measures the construct that this questionnaire intends to measure. For instance, the Social Roles Questionnaire is a well-developed questionnaire, it has two inherent differences with Home and Family Work Roles Questionnaire [32]. For one thing, the Social Roles Questionnaire measures the ‘attitudes’ and not the actual proportion of work done by the partners in a household [32]. Moreover, the Social Roles Questionnaire measures the attitudes towards gendered tasks both inside and outside the household, and applies to highly gendered tasks, whereas the Home and Family Work Roles Questionnaire intends to apply to all activities of the household [32]. The same principles apply to the Attitudes Toward Women Scale and the Personal Attributes Questionnaires [33].

### Limitations

As this study was nested within a larger cross-sectional and anonymous study, our ability to collect data for testing other psychometric properties was limited. For example, even though we reported the internal consistency, we were not able to calculate the test–retest reliability. Further, we were only able to do EFA since no prior data existed on the Home and Family Work Roles Questionnaire. Nonetheless, the EFA results reported in this study are supported by theory. Therefore, future studies are warranted to further approve the three-factor solution proposed in this study by confirmatory factor analysis and structural equation modelling. Finally, our sample had a large percent of women participants and not a sufficient number of other genders to statistically analyze the latter subgroup.

## Conclusion

The Home and Family Work Roles Questionnaire was validated using EFA, and three factors were established, which were Caregiving, Traditionally Feminine, and Traditionally Masculine Roles. Further, excellent internal consistency of the Home and Family Work Roles Questionnaire was established (Cronbach's α = 0.94). We suggest future studies to further probe the recommended factors by conducting confirmatory factor analysis and to assess other important psychometric properties such as the test–retest reliability and content validity.

## Supplementary Information

**Additional file 1.** The descriptive statistics of the Caregiving, Tarditionally Feminine, and Traditionally Masculine factors.

## Data Availability

The datasets generated and/or analyzed during the current study are not publicly available but are available from the corresponding author on reasonable request.
