# Supplementary material for: Construct validity and internal consistency of the Home and Family Work Roles Questionnaires: a cross-sectional study with exploratory factor analysis
Source: BMC Womens Health. 2023 Feb 10;23:56. doi: 10.1186/s12905-023-02199-1 (PMC9911936; doi:10.1186/s12905-023-02199-1)
Supplement: Supplementary file 1 — Additional file 1. The descriptive statistics of the Caregiving, Tarditionally Feminine, and Traditionally Masculine factors. [file 12905_2023_2199_MOESM1_ESM.docx]

**Appendices**

**Appendix I. Descriptives- Caregiving Roles Factors**

|  |  |  |  |  |  |  |  |  |  |  |  |  |  |  |  |
| --- | --- | --- | --- | --- | --- | --- | --- | --- | --- | --- | --- | --- | --- | --- | --- |
|  | | **Gender** | | **Arrange family appointments and activities** | | **Help children with homework** | | **Supervise children with homework** | | **Care for children in the home** | | **Care for children when sick** | | **Care for other family members** |  |
| N |  | Man |  | 43 |  | 42 |  | 41 |  | 44 |  | 41 |  | 40 |  |
|  |  | Woman |  | 254 |  | 238 |  | 233 |  | 259 |  | 247 |  | 204 |  |
| Missing |  | Man |  | 2 |  | 3 |  | 4 |  | 1 |  | 4 |  | 5 |  |
|  |  | Woman |  | 11 |  | 27 |  | 32 |  | 6 |  | 18 |  | 61 |  |
| Mean |  | Man |  | 2.49 |  | 3.29 |  | 3.12 |  | 3.43 |  | 2.80 |  | 2.95 |  |
|  |  | Woman |  | 6.18 |  | 6.26 |  | 6.18 |  | 6.29 |  | 6.44 |  | 4.69 |  |
| Median |  | Man |  | 1 |  | 2.50 |  | 2 |  | 4.00 |  | 3 |  | 2.00 |  |
|  |  | Woman |  | 8.00 |  | 8.00 |  | 8 |  | 8 |  | 8 |  | 5.00 |  |
| Standard deviation |  | Man |  | 2.77 |  | 3.29 |  | 3.25 |  | 2.91 |  | 2.66 |  | 3.05 |  |
|  |  | Woman |  | 4.06 |  | 3.97 |  | 4.07 |  | 3.60 |  | 3.99 |  | 3.58 |  |
| Skewness |  | Man |  | 1.09 |  | 0.67 |  | 0.76 |  | 0.15 |  | 0.27 |  | 0.80 |  |
|  |  | Woman |  | -0.47 |  | -0.58 |  | -0.56 |  | -0.69 |  | -0.67 |  | 0.12 |  |
| Std. error skewness |  | Man |  | 0.36 |  | 0.37 |  | 0.37 |  | 0.36 |  | 0.37 |  | 0.37 |  |
|  |  | Woman |  | 0.15 |  | 0.16 |  | 0.16 |  | 0.15 |  | 0.15 |  | 0.17 |  |
| Kurtosis |  | Man |  | 0.60 |  | -0.76 |  | -0.56 |  | -1.34 |  | -1.19 |  | -0.38 |  |
|  |  | Woman |  | -1.48 |  | -1.31 |  | -1.37 |  | -0.94 |  | -1.20 |  | -1.32 |  |
| Std. error kurtosis |  | Man |  | 0.71 |  | 0.72 |  | 0.72 |  | 0.70 |  | 0.72 |  | 0.73 |  |
|  |  | Woman |  | 0.30 |  | 0.31 |  | 0.32 |  | 0.30 |  | 0.31 |  | 0.34 |  |

**Appendix II. Descriptives- Traditionally Feminine Roles Factor**

|  | | Gender | | House cleaning | | Laundry | | Home decorating | | Prepare meals | | Shop for groceries and supplies | | Drive family to appointments and activities | | Earn family income | | Manage family finances/bills | |
| --- | --- | --- | --- | --- | --- | --- | --- | --- | --- | --- | --- | --- | --- | --- | --- | --- | --- | --- | --- |
| N |  | Man |  | 45 |  | 45 |  | 40 |  | 45 |  | 45 |  | 43 |  | 43 |  | 43 |  |
|  |  | Woman |  | 265 |  | 265 |  | 237 |  | 265 |  | 265 |  | 250 |  | 246 |  | 259 |  |
| Missing |  | Man |  | 0 |  | 0 |  | 5 |  | 0 |  | 0 |  | 2 |  | 2 |  | 2 |  |
|  |  | Woman |  | 0 |  | 0 |  | 28 |  | 0 |  | 0 |  | 15 |  | 19 |  | 6 |  |
| Mean |  | Man |  | 5.04 |  | 3.84 |  | 3.33 |  | 4.38 |  | 4.98 |  | 3.77 |  | 4.51 |  | 3.98 |  |
|  |  | Woman |  | 6.62 |  | 6.91 |  | 5.30 |  | 6.80 |  | 6.27 |  | 5.62 |  | 3.99 |  | 5.04 |  |
| Median |  | Man |  | 5 |  | 3 |  | 2.00 |  | 4 |  | 5 |  | 4 |  | 5 |  | 3 |  |
|  |  | Woman |  | 7 |  | 8 |  | 5 |  | 7 |  | 7 |  | 5.50 |  | 4.00 |  | 5 |  |
| Standard deviation |  | Man |  | 2.66 |  | 3.04 |  | 3.28 |  | 3.02 |  | 3.60 |  | 3.24 |  | 3.95 |  | 3.91 |  |
|  |  | Woman |  | 2.69 |  | 2.98 |  | 3.87 |  | 2.83 |  | 3.55 |  | 3.88 |  | 3.65 |  | 4.04 |  |
| Skewness |  | Man |  | 0.24 |  | 0.64 |  | 0.69 |  | 0.35 |  | 0.14 |  | 0.43 |  | 0.17 |  | 0.43 |  |
|  |  | Woman |  | -0.43 |  | -0.59 |  | -0.09 |  | -0.63 |  | -0.43 |  | -0.23 |  | 0.41 |  | 0.00 |  |
| Std. error skewness |  | Man |  | 0.35 |  | 0.35 |  | 0.37 |  | 0.35 |  | 0.35 |  | 0.36 |  | 0.36 |  | 0.36 |  |
|  |  | Woman |  | 0.15 |  | 0.15 |  | 0.16 |  | 0.15 |  | 0.15 |  | 0.15 |  | 0.16 |  | 0.15 |  |
| Kurtosis |  | Man |  | -0.76 |  | -0.58 |  | -0.73 |  | -0.96 |  | -1.36 |  | -0.81 |  | -1.51 |  | -1.41 |  |
|  |  | Woman |  | -0.83 |  | -0.94 |  | -1.57 |  | -0.62 |  | -1.30 |  | -1.52 |  | -1.19 |  | -1.63 |  |
| Std. error kurtosis |  | Man |  | 0.69 |  | 0.69 |  | 0.73 |  | 0.69 |  | 0.69 |  | 0.71 |  | 0.71 |  | 0.71 |  |
|  |  | Woman |  | 0.30 |  | 0.30 |  | 0.31 |  | 0.30 |  | 0.30 |  | 0.31 |  | 0.31 |  | 0.30 |  |
|  | | | | | | | | | | | | | | | | | | | |

**Appendix III. Descriptives- Traditionally Masculine Role Factor**

|  |  |  |  |  |  |  |  |  |  |  |  |  |  |
| --- | --- | --- | --- | --- | --- | --- | --- | --- | --- | --- | --- | --- | --- |
|  | | **Gender** | | **Outdoor cleaning** | | **Home repairs** | | **Mow lawn** | | **Garden** | | **Maintain vehicles** | |
| N |  | Man |  | 45 |  | 44 |  | 42 |  | 43 |  | 42 |  |
|  |  | Woman |  | 260 |  | 259 |  | 214 |  | 251 |  | 253 |  |
| Missing |  | Man |  | 0 |  | 1 |  | 3 |  | 2 |  | 3 |  |
|  |  | Woman |  | 5 |  | 6 |  | 51 |  | 14 |  | 12 |  |
| Mean |  | Man |  | 5.49 |  | 5.48 |  | 5.98 |  | 3.79 |  | 5.98 |  |
|  |  | Woman |  | 3.99 |  | 3.27 |  | 2.01 |  | 4.96 |  | 2.92 |  |
| Median |  | Man |  | 6 |  | 7.00 |  | 8.00 |  | 3 |  | 7.50 |  |
|  |  | Woman |  | 3.00 |  | 2 |  | 0.00 |  | 5 |  | 1 |  |
| Standard deviation |  | Man |  | 3.22 |  | 3.97 |  | 4.33 |  | 3.35 |  | 3.85 |  |
|  |  | Woman |  | 3.16 |  | 3.19 |  | 3.16 |  | 3.65 |  | 3.30 |  |
| Skewness |  | Man |  | -0.21 |  | -0.28 |  | -0.44 |  | 0.52 |  | -0.49 |  |
|  |  | Woman |  | 0.60 |  | 0.87 |  | 1.57 |  | 0.09 |  | 0.95 |  |
| Std. error skewness |  | Man |  | 0.35 |  | 0.36 |  | 0.37 |  | 0.36 |  | 0.37 |  |
|  |  | Woman |  | 0.15 |  | 0.15 |  | 0.17 |  | 0.15 |  | 0.15 |  |
| Kurtosis |  | Man |  | -1.11 |  | -1.63 |  | -1.61 |  | -1.00 |  | -1.32 |  |
|  |  | Woman |  | -0.84 |  | -0.45 |  | 1.19 |  | -1.41 |  | -0.36 |  |
| Std. error kurtosis |  | Man |  | 0.69 |  | 0.70 |  | 0.72 |  | 0.71 |  | 0.72 |  |
|  |  | Woman |  | 0.30 |  | 0.30 |  | 0.33 |  | 0.31 |  | 0.31 |  |
